# Thymus Reconstitution in Young and Aged Mice Is Facilitated by *In Vitro*-Generated Progenitor T Cells

**DOI:** 10.3389/fimmu.2022.926773

**Published:** 2022-07-08

**Authors:** Mahmood Mohtashami, Yue Ru Li, Christina R. Lee, Juan Carlos Zúñiga-Pflücker

**Affiliations:** ^1^ Biological Sciences, Sunnybrook Research Institute, Toronto, ON, Canada; ^2^ Department of Immunology, University of Toronto, Toronto, ON, Canada

**Keywords:** hematopoietic stem cell (HSC) transplantation, progenitor T cells, T cell development, thymus, homing

## Abstract

The prolonged lag in T cell recovery seen in older patients undergoing hematopoietic stem cell transplant (HSCT), after chemo-/radiotherapy, can lead to immune dysfunction. As a result, recovering patients may experience a relapse in malignancies and opportunistic infections, leading to high mortality rates. The delay in T cell recovery is partly due to thymic involution, a natural collapse in the size and function of the thymus, as individuals age, and partly due to the damage sustained by the thymic stromal cells through exposure to chemo-/radiotherapy. There is a clear need for new strategies to accelerate intrathymic T cell reconstitution when treating aged patients to counter the effects of involution and cancer therapy regimens. Adoptive transfer of human progenitor T (proT) cells has been shown to accelerate T cell regeneration in radiation-treated young mice and to restore thymic architecture in immunodeficient mice. Here, we demonstrate that the adoptive transfer of *in vitro*-generated proT cells in aged mice (18-24 months) accelerated thymic reconstitution after treatment with chemotherapy and gamma irradiation compared to HSCT alone. We noted that aged mice appeared to have a more limited expansion of CD4-CD8- thymocytes and slower temporal kinetics in the development of donor proT cells into mature T cells, when compared to younger mice, despite following the same chemo/radiation regimen. This suggests a greater resilience of the young thymus compared to the aged thymus. Nevertheless, newly generated T cells from proT cell engrafted aged and young mice were readily present in the periphery accelerating the reappearance of new naïve T cells. Accelerated T cell recovery was also observed in both aged and young mice receiving both proT cells and HSCT. The strategy of transferring proT cells can potentially be used as an effective cellular therapy in aged patients to improve immune recovery and reduce the risk of opportunistic infections post-HSCT.

## Introduction

T cells play a key role in the adaptive immunity to protect individuals from infections and malignancies. Most of the events during T cell development occur within the thymus, which is a primary immune organ that normally lacks self-renewing T cell progenitors (1). Therefore, the thymus relies on the semicontinuous supply of thymus-seeding progenitors (TSPs) from the bone marrow (BM) (2). TSPs receive strong Notch signals in the thymus (3), which guides them through a series of regulated developmental steps, including CD4^-^ CD8^-^ double negative (DN), CD4^+^ CD8^+^ double positive (DP), and CD4^+^ or CD8^+^ single positive (SP) stages (4). TSPs commit to the T-lineage during intrathymic differentiation and become mature T cells when receiving signals from the specialized thymic microenvironment (5).

Interventions that disrupt the generation of T cells can lead to immunodeficiencies, such as irradiation/chemotherapy required for the treatment of some cancers (6, 7). Clinically, patients with hematological malignancies require myeloablative chemotherapy and/or radiotherapy to eliminate leukemic cells followed by hematopoietic and stem cell transplant (HSCT) from compatible donors to reconstitute the depleted BM niche. Although most blood borne cells recover relatively quickly, T cells have a prolonged recovery period, leading to higher risks of opportunistic infections or relapse (8). This is likely due by the disrupted process of lymphopoiesis as a result of irradiation and chemotherapy (*e.g.*, cyclophosphamide), particularly due to damage to thymic epithelial cells (TECs) (9, 10).

The delay in T cell recovery, which can last for over a year, is exacerbated in elderly patients (11), largely as a result of thymic involution, the natural age-related atrophy of the T-lymphopoietic organ. This has been modeled by showing that TECs in aged mice showed a higher rate of apoptosis and lower proliferative capacity (12), which was concomitant with a decline in the expression of the TEC-specific master transcription factor, Forkhead box N1 (FOXN1) (13, 14). In addition, aged thymus has a deficiency in the enzyme catalase, leading to an accumulation of damaging reactive oxygen species in TECs (15–17). The aged thymus also has a disorganized thymic structure, with a thinning of cortical regions, and more adipocytes and fibroblasts, providing a suboptimal microenvironment for the development and survival of proT cells (18, 19). This is further exacerbated by the absence of thymocyte/TEC crosstalk following HSCT, which may include RANK stimulation, required for proper thymic maintenance (20–22), potentially adding to the decline of the aged thymus compared to young (23).

A potential strategy to circumvent the paucity in T cell regeneration seen with HSCT is to adoptively transfer *in vitro*-generated progenitor T (proT) cells along with HSCT to facilitate thymic engraftment and accelerate T cell reconstitution (23–25). We define mouse proT cells as CD25^+^ DN cells (DN2 and DN3 stages) that can home to thymus, while possessing limited potential for non-T lineages outcomes. ProT cells have been shown to effectively reconstitute a host thymus and differentiate into all T cell subsets. Since donor proT cells undergo positive- and negative-selections in the host thymus, newly generated T cells are then restricted to host self-MHC, and tolerized to self-antigens, free from the risk of graft-vs-host-disease (26). Additionally, we have shown that through lymphocyte/TEC crosstalk, proT cells improve the thymic architecture of immunodeficient mice and enhance subsequent recruitment of bone marrow-derived progenitors (27). However, the kinetics of thymus recovery after combined therapy of proT cell and HSCT in aged mice remained unknown.

Here, we show that co-administration of *in vitro*-generated proT cells and HSCT can accelerate thymic reconstitution in aged and young mice after chemotherapy and radiotherapy, as compared to HSCT alone. Furthermore, we demonstrate that aged mice receiving proT cells have accelerated T cell recovery in the periphery, as compared to mice given HSCT alone. Notably, proT cells showed a similar ability to home to the thymuses of aged and young mice. However, aged recipient hosts showed a more limited expansion of donor thymocytes and slower kinetics of T cell development as compared to the young mice. Our preclinical results confirm that co-transferring proT cells with HSCT can potentially be used as an effective cellular therapy to enhance the immune recovery and lower the risk of opportunistic infections in aged patients post-HSCT.

## Methods

### Mice

C57BL/6 (B6 CD45.2) and congenic B6.SJL-Ptprca Pepcb/BoyJ (B6 CD45.1) mice were purchased from The Jackson Laboratory (stock numbers 000664 and 002014, respectively). Young (8 to 12 weeks), and in-house aged (18-20 months) cohorts of mice were used. Green fluorescent protein (GFP^+^) hematopoietic cells were generated by breeding ROSA26-rtTA transgenic mice (3) to Vav-iCre transgenic mice (4) to establish VaviCre-ROSA26rtTA mice on the B6 CD45.2 background. In hematopoietic cells, GFP was expressed upon Cre-dependent removal of a loxP-stop-loxP cassette within the ROSA26 locus. DsRed (B6.Cg-Tg(CAG-DsRed*MST)1Nagy/J) transgenic mice were purchased from The Jackson Laboratory (stock number 006051). All mice were maintained and bred at Sunnybrook Health Sciences Centre, and all animal procedures were approved by the Sunnybrook Health Sciences Centre Animal Care Committee.

### Progenitor T Cell Co-Cultures With OP9-DL4-7FS Cells

Lineage-negative (Lin^-^) Sca-1^+^ Kit^+^ (LSK)/OP9-DL4 cell co-cultures were implemented, as previously described (5) with several modifications. Briefly, we cultured mouse-BM-derived LSK cells with the newly generated OP9-DL4-7FS cell line, transduced to express the Notch ligand *Dll4* as well as human cytokines IL-7, FLT3-L, and SCF, as described (28). BM cells were collected from wild type B6 mice by dissecting and crushing the leg bones using sterile utensils in Hanks’ Balanced Salt Solution (HBSS). BM cells were then filtered through 40 µm filter to get a single cells suspension. CD117^+^ (Kit^+^) cells were enriched using anti-CD117-MicroBeads and LS column (Miltenyi) according to manufacturer’s instructions. Subsequently, the CD117-enriched population was labelled with FITC-conjugated antibodies against lineage (Lin) markers [anti-B220 (RA3–6B2), anti-CD19 (1D3), anti-CD11b (M1/70), anti-Gr-1 (8C5), anti-NK1.1 (PK136), anti-CD3 (2C11), anti-CD8α (53.6–7), anti-CD4 (GK1.5)], as well as with anti-CD117-APC (2B8) and anti-Sca1-PE (D7) (all from BioLegend). LSK cells were sorted using cell sorter FACSAria Fusion (BD Biosciences). In experiments using LSK cells from DsRed^+^ donor mice, the same procedure was performed with Sca-1 coupled to APC-Cy7 fluorophore (BioLegend).

In each 15 cm culture dish of OP9-DL4-7FS cells at ~90% confluency, 50,000 to 70,000 LSK cells were seeded and maintained in α-Minimum Essential Medium Eagle (α-MEM) supplemented with 5% FBS and 1% Penicillin/Streptomycin (Gibco) in the presence of 1 ng/ml IL-7 (Miltenyi Biotec) and 5 ng/ml Flt-3L (Miltenyi Biotec). Old culture media was ½ replaced with fresh media with no additional cytokines on days 5 and 8 after the start of co-cultures. On day 10 after seeding the LSK cells, the co-cultures were harvested and filtered through 40 µm cell strainers (Thermo-Fisher). The single cell suspension was labelled with anti-CD25-APC (PC61, Bio-Legend) and subsequently incubated in anti-APC-MicroBeads (Miltenyi Biotec), and enriched for CD25^+^ cells using LS column (Miltenyi Biotec) according to manufacturer’s instructions. The flow through (CD25^-^ cells) was also collected for injection in some experiments.

### Adoptive Transfer of Progenitor T Cells

B6 CD45.2 or CD45.1 congenic hosts were IP-injected with 150 µg/kg of Cyclophosphamide (Procytox (CTX), Baxter Corp.) 5 days and 3 days prior to exposure to 1.05 Gy total body irradiation using a Cs137 source gamma irradiator. 4-6 hours post-irradiation, all mice were intravenously injected with 1×10^6^ B6 GFP^+^ BM-extracted cells from VaviCre-ROSA26rtTA mice that were T cells depleted (T-depleted Bone Marrow, TDBM). T cells in the BM VaviCre-ROSA26rtTA mice were depleted by anti-CD3-MicroBeads (Miltenyi). The “proT+TDBM” experimental group also received 5×10^5^, 1×10^6^, 2×10^6^, or 4×10^6^ CD25-enriched proT cells derived from B6 CD45.2 or CD45.1 mice, at 99% purity. Cells were resuspended in 200 µL of serum-free αMEM in preparation for injections. In the CD25^+^ vs. CD25^-^ experiment, each experimental group was intravenously injected with 5×10^5^ CD25^+^ or CD25^-^ cells.

### Flow Cytometric Analysis

Single cell suspensions of dissected thymus, spleen, and BM were prepared by mashing followed by filtering through 40 µL cell strainers in HBSS supplemented with 1% bovine serum albumin and 2 mM EDTA. Single-cell suspensions were labelled with fluorescently-conjugated antibodies purchased from BioLegend as follows: CD45.1(A20)-Percp/Cy5.5, CD45.2(104)-APC/Cy7, CD4(GK1.5)-Alexafluor 700, CD8(53.6–7)-PE/Cy7, CD44(IM7)-PE, CD25-APC, CD3(17A2)-PE/Cy7, CD11b(M1/70)-APC, CD19(1D3)-PE, CD45(30F-11)-APC/Cy7. Flow cytometry was performed on LSR II (BD Biosciences). Dead cells were excluded by 4′,6-diamidino-2-phenylindole (DAPI) uptake. Data were analyzed using FlowJo Version 10.8.1 software (TreeStar).

### Statistical Analysis

Statistically significance between different adoptive transfer dosages of proT cells and between aged and young mice in their thymuses and spleens were analyzed using one-way or two-way ANOVA and were performed using Prism software. All data are represented as mean ± SEM in error bars, with asterisks representing statistical significance (*p < 0.05, **p < 0.01, ***p < 0.001, ****p < 0.0001).

## Results

### Available Thymic Niches Are Similar in Young and Aged Mice

**Figure 1:**
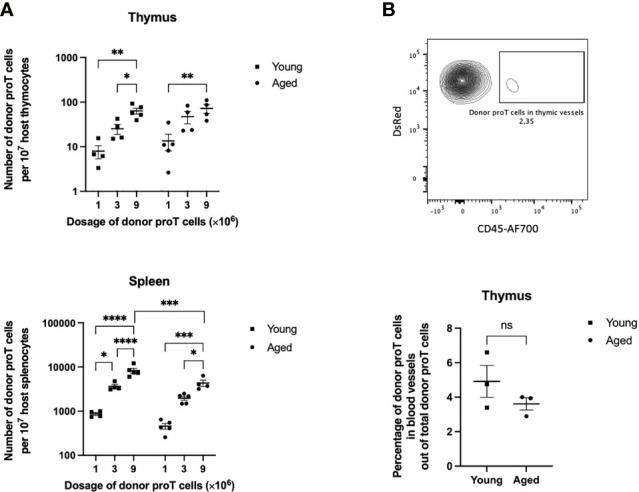
Thymus engraftment by *in vitro*-generated progenitor T cells in unmanipulated young and old mice. **(A)** CD25^+^ DsRed^+^ donor proT cells per 10^7^ host thymocytes (top) and splenocytes (bottom) isolated from host thymuses and spleens of non-irradiated aged and young mice. Each group of mice (n=4-5) were injected with 1, 3, or 9 x10^6^ proT cells. Error bars depict SEM; *p<0.05, **p<0.01, ***p<0.001, ****p<0.0001 analyzed by two-way ANOVA. **(B)** Representative flow cytometric analysis of detected DsRed^+^ proT cells from the thymus of a host mouse (top). Aggregated percentages of DsRed^+^ proT cells in blood vessels out of total DsRed^+^ proT cells from host thymuses injected with either 1, 3, or 9 x10^6^ proT cells (bottom). Hosts were intravenously injected with CD45-AF700 antibodies 3 minutes prior to sacrificing. Error bars depict SEM; difference between the aged and young mice groups was not significantly (ns) different as analyzed by two tailed unpaired student’s *t*-test.

To determine whether the thymuses of aged and young mice have an intrinsically different capacity to recruit proT cells and/or a different number of available thymic niches under steady state conditions, we adoptively transferred increasing numbers of *in vitro*-generated proT cells into non-irradiated mice in each age group (**Figure 1**). To this end, sorted mouse bone marrow (BM) Lineage^-^ Sca-1^+^ Kit^+^ (LSK) cells were cocultured with OP9-DL4-7FS cells (28) for 10 days to generate CD25^+^ proT cells at the DN2/DN3 stage of T cell development (**Supplementary Figure 1A**). Cocultures were subjected to magnetic-assisted cell sorting (MACS) to enrich for CD25^+^ and the CD25^-^ subsets.

CD25^+^ proT cells (1, 3, or 9 x10^6^ CD25^+^ cells), *in vitro*-generated from LSK cells from DsRed mice, were injected intravenously (i.v.) into host mice, and the presence of donor cells within the thymus was assessed at 40 h after injection. To rule out proT cells outside the thymic parenchyma, a labelled anti-CD45 antibody was injected i.v. just prior to sacrificing the host mice. Flow cytometric analysis of thymuses from young mice revealed the presence of donor DsRed^+^ cells when injected with 1 x10^6^ proT cells, and the numbers of donor cells appeared to increase linearly when 3 and 9 x10^6^ donor proT cells were adoptively transferred (**Figure 1A**). However, the thymus of aged mice showed saturated niches with increasing numbers of injected proT cells. Of note, the frequency of proT cells present outside the thymic parenchyma, or perivascular space, was on average ≤5% in both aged and young mice (**Figure 1B**). Surprisingly, the thymus of aged mice showed a similar capacity in recruiting donor proT cells, with the thymus of young and aged mice failing to show a significantly different number of receptive niches. Similar to the thymus, host spleens showed a dose-dependent appearance of donor DsRed^+^ cells. However, the number of donor cells present in spleens was over 40-fold higher than what was detected in the thymus. This suggested that at steady state the thymus has a highly restricted entry and/or limited number of niches for proT cells.

### Thymus Engraftment by *In Vitro* Generated ProT Cells

**Figure 2:**
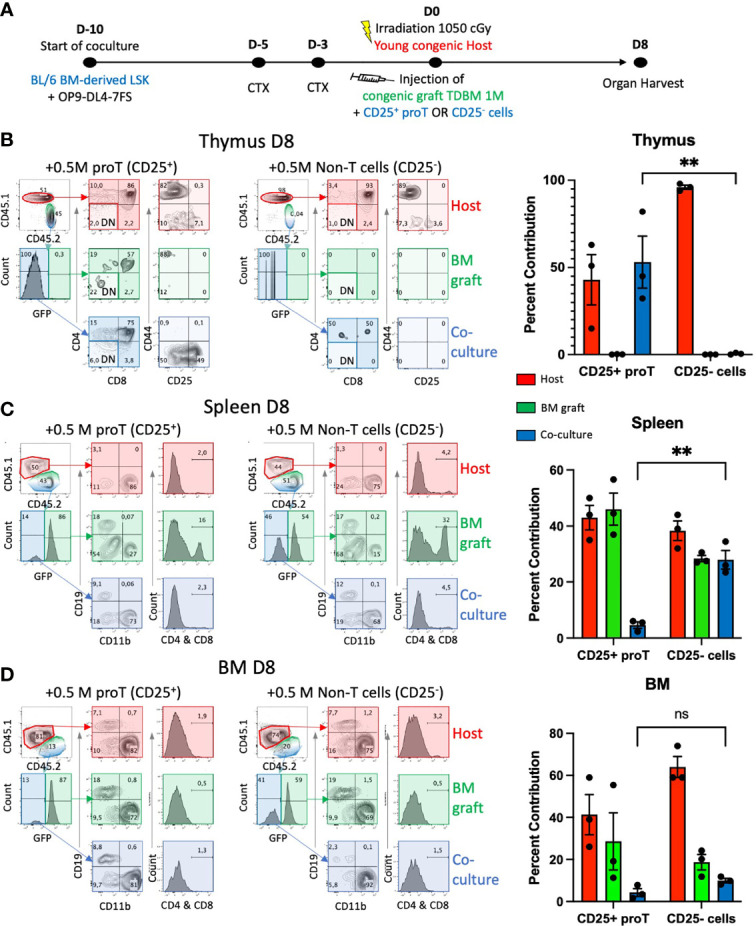
Thymus engraftment by *in vitro-*generated proT cells. **(A)** Experimental schematic for generating proT cells in the HSC/OP9DL47FS system. BM-derived LSK cells from CD45.2 mice were obtained and co-cultured with OP9-DL4-7FS cells 10 days prior to injection. Young (8-12 wks) B6 CD45.1 mice were intraperitoneally injected with cyclophosphamide (CTX) 5 days and 3 days prior to injection and lethally irradiated at 1.05 Gy on the day of injection. All mice were intravenously injected with 1 x10^6^ hematopoietic cells extracted from the BM of CD45.2 GFP^+^ mice that were T cell-depleted (TDBM cells) and 5 x10^5^ CD25^+^ or CD25^-^ culture-derived cells. On D8 post injection, graft and host contribution to the host **(B)** thymus, **(C)** spleen, **(D)** BM, were analyzed by flow cytometry. In **(B)**, CD44 vs. CD25 panels were gated on CD4- CD8- DN cells (lighter shade for DN-gated panels). For each organ, the contribution of host (red), GFP^+^ BM graft (green) or coculture derived CD25^+^ or CD25^-^ cells (blue) were calculated and graphed. Significant difference between coculture-derived cells was noted (**p<0.01 analyzed by two-way ANOVA, error bars depict SEM), ns, not significant.

The use of non-irradiated host mice established that the thymus of both young and aged mice showed a similar but low number of receptive niches. We then assessed whether the use of clinically relevant conditioning regimens, including chemo/radiotherapy, would impact the effectiveness of proT cell engraftment in aged and young mice. To this end, young host mice were treated with cyclophosphamide (CTX, 150 µg/kg) 5 and 3 days prior to lethal irradiation (1.05 Gy) (**Figure 2A**). To verify that *in vitro*-generated proT cells and no other cocultured-derived cells would home to the thymus, as described before (24, 25) and shown above, we compared the homing and engraftment ability of LSK/OP9-DL47FS coculture-generated CD25^+^ DN proT cells to that of the remaining CD25^-^ DN cells (**Supplementary Figure 1A**). In addition to the culture-derived cells, young mice (8-12 wks old) were co-injected with 1 x10^6^ GFP^+^ T cell-depleted BM (TDBM) cells. The thymuses of host mice were analyzed by flow cytometry 8 days (D8) after injection, and we noted a significantly greater number of donor CD25^+^ proT cells homing to and developing within the thymus, constituting ~50% of total thymocytes, including CD4^+^ CD8^+^ DP cells, in contrast to CD25^-^ DN cells, which failed to engraft (**Figure 2B**). In this regard, mice injected with CD25^-^ DN cells showed only host-derived cells in the thymus (**Figure 2B**). Remarkably, on D8, some of the CD25^+^ proT-derived thymocytes still exhibited CD25 expression, suggesting long-lasting self-renewal of proT cells within the thymus (**Figure 2B**, left panel). Nevertheless, mice injected with CD25^-^ DN cells showed a minor fraction (~0.5%) of donor cells within the thymus that progressed towards the DP stage of differentiation, which we attributed to the fact that the MACS-enriched CD25^-^ DN population contained about 8% CD25^+^ proT cells (**Supplementary Figure 1A**, middle panel). In contrast to the poor thymic engraftment, CD25^-^ DN cells were readily detected in the spleen of host mice, comprising the 28% of splenocytes, with the vast majority expressing CD11b (**Figure 2C** and **Supplementary Figure 1B**). As expected, the other major contributor to CD11b^+^ myeloid cells in the spleen are derived from the GFP^+^ TDBM donor graft. Remarkably, the contribution of culture-derived donor cells to the BM was minimal for both CD25^+^ proT and CD25^-^ DN cells, while as expected a strong contribution by GFP^+^ TDBM donor cells was observed (**Figure 2D**). Taken together, our findings further validate the use of *in vitro*-generated CD25^+^ proT cells as an effective thymus seeding cell in the context of HSCT.

### Thymus Engraftment by Increasing Numbers of ProT Cells in Aged Mice

**Figure 3:**
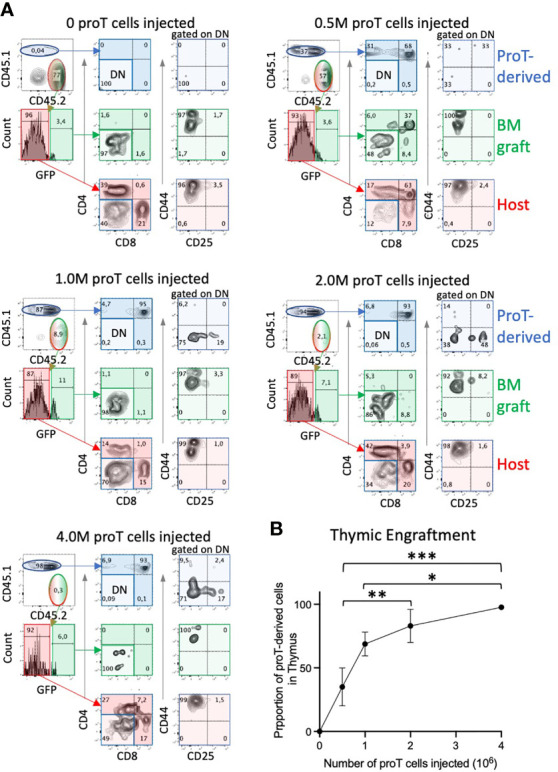
Thymic engraftment of aged mice is dependent on proT cell dosage. Conditioned aged B6 CD45.2 mice were i.v. injected with 1 x 10^6^ TDBM from CD45.2 GFP^+^ mice and an increasing number of proT cells, *in vitro*-generated from CD45.1 mice, ranging from 0.5, 1, 2 and 4 x10^6^. Thymuses were harvested on D14 and assessed for the presence of T cell markers using flow cytometry. Representative plots are shown in **(A)**. CD44 vs. CD25 panels were gated on CD4^-^ CD8^-^ DN cells. Percentage of thymic cells derived from CD45.1^+^ proT cells are shown in graph in **(B)** (n=4; error bars depict SEM; *p<0.05, **p<0.01, ***p<0.001. analyzed by two-way ANOVA). ns, not significant.

To determine the number of proT cells required to saturate thymic engraftment in aged mice, in the context of combined chemo/radiation conditioning and HSCT, we adoptively transferred 0, 0.5, 1, 2 or 4 x10^6^ congenic *in vitro*-generated proT cells along with 1 x10^6^ GFP^+^ TDBM cells into aged mice, as illustrated in **Figure 2A**. On D14, we used flow cytometry to determine the contribution of host, GFP^+^ TDBM and proT cells to thymus cellularity (**Figure 3**). All aged mice receiving proT cells showed the presence of donor-derived DP cells by D14. Mice injected with only TDBM (0 proT) did not show the presence of DPs, either host or TDBM derived, rather contained host CD4 or CD8 SP T cells and very few, if any, TDBM derived cells, which were nearly all at the DN stage. There was a positive correlation between engraftment efficiency and the number of proT cells injected (**Figure 3B**), with the provision of 4 x10^6^ cells resulting in nearly 100% of thymic cellularity corresponding to proT-derived cells. This suggested that all the possible niches were likely occupied by the injection of 4 x10^6^ proT cells, competing out host and GFP^+^ TDBM donor cells to exclusively participate in early thymic engraftment.

### ProT Cells Accelerate Thymus Engraftment in Both Young and Aged Mice

**Figure 4:**
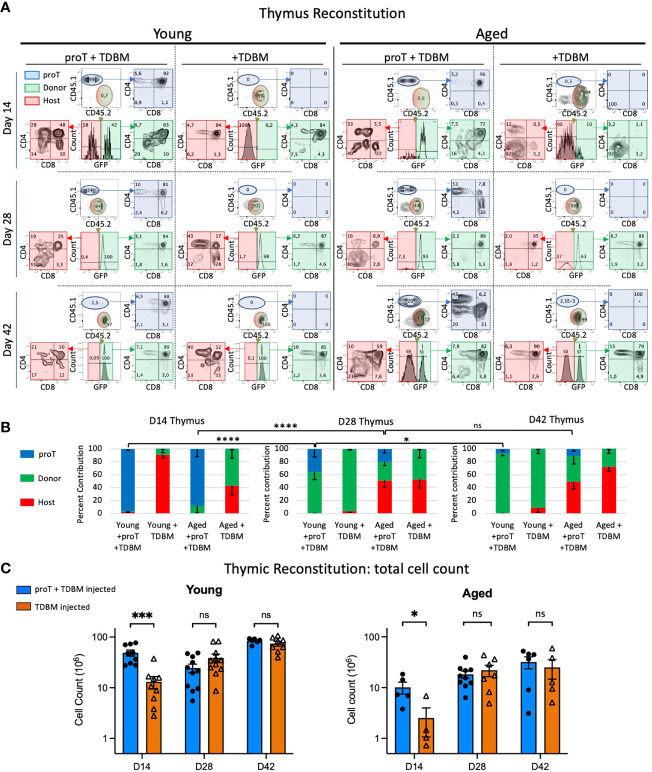
Thymic engraftment of young and aged mice. Young (8-12wks) and aged (18-20 months) mice were treated with CTX followed by lethal irradiation in preparation for i.v. injection of either 4 x 10^6^ proT cells derived from LSK/OP9-DL4 cocultures plus 1 x10^6^ TDBM or 1 x10^6^ TDBM alone as indicated. Thymus and other organs were harvested on days 14 (D14), D28 and D42. Thymocytes were labelled with appropriate lineage markers and analyzed by flow cytometry represented by plots shown in **(A)**. The thymocyte population was contributed by cells derived from the host (red), GFP^+^ BM graft (green) and proT coculture (blue). The percent contribution of the source, whether host (CD45.2^+^, GFP^-^), BM graft-derived (CD45.2^+^, GFP^+^) or proT-derived (CD45.1^+^) is shown in **(B)** for the three timepoints. Total cell number of thymocytes is depicted in **(C)** with young in the left panel and the aged in the right panel. For all samples at all time points, n≥4 up to 12. Error bars indicated SEM as indicated in *Methods*; significance was measured with two-way ANOVA. *p<0.05, ***p<0.001, ****p<0.0001.

Having established the number of proT cells required to fully engraft the thymus in aged mice, we then compared proT cell engraftment and differentiation in aged vs. young mice. We analyzed host mice, young and aged, in 2-week intervals following the administration of 4 x10^6^ proT cells and 1 x10^6^ GFP^+^ TDBM cells (**Figure 4A**). On D14, proT-derived cells constitute ~95% of thymocytes present in young mice and ~87% in aged mice (**Figure 4B**), with most cells (~90%) having reached the CD4/CD8 DP stage of differentiation in both young and aged thymuses. Of note, a major difference between young and aged host mice was the number of thymocytes after engraftment, with thymus of young mice having ~5-fold higher total cellularity than aged mice at D14 (**Figure 4C**). In contrast, mice receiving only TDBM cells showed a delayed thymic engraftment. Importantly, at D14, TDBM mice had ~4-fold fewer thymocytes when compared to their age-matched mice that had also received proT cells, confirming that proT cells significantly enhance thymic reconstitution in both young and aged mice.

*In vitro* generated proT cells may represent a finite source of thymic seeding cells, after entering the thymus and differentiating into later stages of T cell development. However, whether proT cells could undergo limited self-renewal after thymic entry was addressed by establishing whether proT-derived short lived DP cells, as well as SP cells, could be seen at later time points. We noted an increase in the proportion of proT-derived SPs in both young and aged thymus by day 28 (**Figure 4A**). However, only the thymuses of young mice revealed the presence of a large percentage of proT-derived DP cells at D28. Of note, even as late as D42, proT-derived DP cells were still detected in the thymus of young mice, albeit coming from a much-reduced frequency of proT-derived cells.

By day 28, the next wave of TSPs derived from the GFP^+^ TDBM cells becomes apparent. We observed a significant decline in the percentage of total thymocytes that were derived from proT cells in young mice, from 96% by D14 to an average of 36% by D28 and only 7% by D42 (**Figure 4B**). This was concomitant with a shift towards an increase in GFP^+^ donor-derived cells within the thymus of young mice (**Figure 4B**). Additionally, total thymus cellularity in young mice, reached ~84 x10^6^ cells by D42, which is similar to unmanipulated aged-matched mice (**Figure 4C**). Remarkably, we noted a dip in thymocyte cellularity at D28 in young mice receiving proT cells (**Figure 4C**), which though statistically not significant, it appears to correspond to the transition from proT-derived cells being the major contributor of thymic cellularity to GFP^+^ donor-derived cells taking over, with over 90% derived from GFP^+^ donor cells (**Figure 4B**).

In aged mice, the frequency of proT-derived thymocytes also significantly declined in percent contribution over time, as these cells are replaced by donor GFP^+^ cells, but also by host cells as well; this latter occurrence was not as readily observed in young mice (**Figure 4**). On D28, both proT- and TDBM-injected aged mice host cells contributed to about 50% of the thymus cellularity, in contrast to less than 5% host contribution in young mice (**Figure 4B**). A similar trend was observed on D42, with the thymuses of aged mice showing a large fraction of host-derived cells irrespective of whether they received proT or TDBM GFP^+^ donor cells.

### Accelerated Peripheral T Cell Reconstitution in Aged ProT-Treated Mice

**Figure 5:**
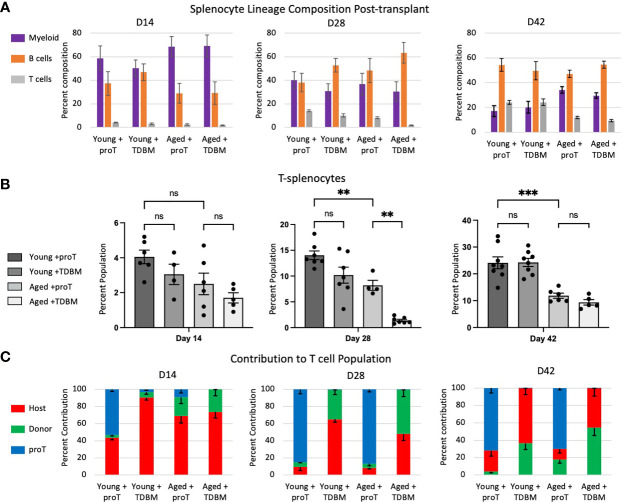
Cellular composition of the spleen post proT adoptive transfer. Mice were treated as indicated in **Figure 4**. Spleens dissected from these mice were processed into single cells and their lineage composition of myeloid (CD11b^+^), B cell (CD19^+^) and T cells (gated first on CD11b^-^ and CD19^-^ population and then on CD3^+^ or CD4^+^ and CD8^+^) was determined through flow cytometry **(A)**. In **(B)**, only the T cell percentage is shown for direct comparison. **(C)** We determined which origin T splenocytes are derived by flow cytometry and expression of CD45.2^+^ GFP^-^ (Host, red), CD45.2^+^ GFP^+^ (Donor, green) and CD45.1^+^ (proT, blue). Error bars indicated SEM. Statistical significance was determined using one-way ANOVA, **p < 0.01, ***p < 0.001.

We next examined whether the rapid thymic engraftment observed in proT-injected mice had an effect on the appearance of peripheral T cells in the spleen. Mice receiving chemo/radio conditioning treatment showed altered immune cell subset distribution in their spleens. While there is variation amongst mouse strains, as well as effects of ageing on the cellular composition of spleen in control unmanipulated mice (29, 30), we observed on average 62% B cells, 25% T cells, 7% myeloid cells and 6% other cells in C57BL/6 spleen. In contrast, on D14 following conditioning, myeloid cells made up the majority of the splenocytes in young or aged mice, whether given proT cells or GFP^+^ TDBM cells (**Figure 5A**). By D28, the proportion of B cells began to recover and approached normal levels, which were reached by D42, and by this time point the proportion of myeloid, B and T cells had returned to control levels.

Focusing on T cells, we noted that the frequency of T cells remain low at D14, both in young and aged mice (**Figure 5B**). By D28, a clear recovery in the percentage of T cells was observed in young mice that were given either proT or TDBM cells, with an increase in T cells to ~14% and ~10%, respectively. Aged mice given proT cells showed an equal recovery, with an increase in T cells to ~8%; whereas, aged mice given TDBM cells failed to recover the proportion of T cells, and remained at less than 2% (**Figure 5B**). This suggests that aged mice given TDBM remain T cell deficient for a significantly longer period when compared to proT-injected aged mice. Nevertheless, by D42 we observed the recovery of T cells in TDBM-injected aged mice.

In terms of the contribution of host, GFP^+^ TDBM or proT-derived cells to splenic T cell population, we found that for proT-injected mice, whether young or aged, the majority of T cells present on D28 were derived from proT cells (**Figure 5C**). This trend continued up to D42, even as the frequency of GFP^+^ TDBM- and host-derived T cells increased, but remained a minority. For young and aged mice given TDBM, there was a trend towards an increased contribution of GFP^+^ donor cells over host cells at all time points. Taken together, these findings support the notion that aged mice given proT cells exhibit an earlier recovery of their T cell compartment.

## Discussion

One of the clinical shortcomings of HSCT is the extended period of time needed for the T cell compartment to reemerge, leading to increased susceptibility to infections and relapse. This is exacerbated in elderly patients (≥60 years old) that comprise the majority of individuals undergoing HSCT for leukemia (6, 31, 32). Adoptive transfer of proT cells into patients has been proposed as a viable future option to combat the prolonged paucity of T cells, since preclinical studies showed that proT cells, generated in culture from HSPCs, can engraft the thymus and accelerate recovery of functional T cells (24, 25). In this study, we addressed whether proT cells can function in a similar manner when given to aged mice, as a preclinical model for elderly patients receiving HSCT. To this end, we also included the administration of cyclophosphamide, a chemotherapy reagent, to better simulate conditioning regimens. Additionally, we expand the pool of proT cells to include all CD3^-^CD4^-^CD8^-^ (DN) CD25^+^ cells, based on the observations that both DN2 and DN3 can engraft the thymus (24).

One of the issues with performing irradiation as the sole approach in mouse models, is that it does not reflect the reality of most clinical modalities, where multiple regimens of chemo- and radio- treatments may be combined. There is also the issue of early auto-reconstitution of host thymocytes by the endogenous radioresistant DN2 subset, which reduces the availability of niches for thymic seeding cells (23). In our model and clinically, as radioresistant DN cells expand and differentiate to repopulate the thymus affected by chemotherapy, these cells are exposed to irradiation, leading to a reduction in the pool of radioresistant DN cells, and potentially less competition for incoming adoptively transferred proT cells. This notion is reflected in the reduction in the requirement of the number of proT cells, from 6-10 x10^6^ (23, 25, 33) to 4 x10^6^, to reach saturation of thymic niches, and with a concomitant increase in the average proportion of proT-derived thymocytes to ~90% within two weeks after adoptive transfer.

Consistent with previous results (12), engraftment of proT cells into the thymus of aged mice appeared to be very similar in efficiency as that seen in young mice, when comparing the number of incoming proT cells within 40 hours after adoptive transfer in absence of conditioning. We extended our analysis to young and aged mice receiving chemo/radio-conditioning and noted by day 14, the total cellularity of the thymus in young mice was 5-fold greater than that obtained in aged mice. This difference in cellularity is likely due to thymic involution, the natural age-related atrophy of the thymus, limiting the niches available for expansion (34). Our results confirmed the likelihood of limiting or poor-quality niches within the thymus of aged mice, affecting the proliferation of donor-derived thymocytes (12, 35).

In particular, we noted that, in the thymus of young mice, short-lived immature DP cells were still being generated from proT cell grafts even after one month since their adoptive transfer. Strikingly, this capacity of proT cells to give rise to DPs for such an extended period after transplant was not seen in the thymus of aged mice. These findings point to a major difference between the thymic microenvironment of young and aged mice, such that proT-derived cells were able to give rise to short-lived DPs for a much longer period of time within the young thymus than when seeding the thymus of aged mice, suggesting that their ability to undergo self-renewal was severely limited within the older thymic microenvironment. Understanding what these deficiencies are within the aged thymic niche will provide important insights as to how to improve T-lymphopoiesis in the elderly.

Despite the known age-associated decrease in thymic output (36, 37), we show here that the thymus of aged mice can export newly generated T cells to the periphery, as detected in the spleen by D28. Of importance, with respect to our modeling, we replicated the lag in the reemergence of peripheral T cells in aged mice receiving TDBM only, when compared to mice given both proT cells and TDBM cells, which showed an earlier appearance of donor-derived T cells in the periphery. We postulate that the aged mice with delayed T cell recovery would be more susceptible to infections, similar to what is seen in elderly patients receiving HSCT. While peripheral T cell reconstitution is accelerated by the provision of proT cells, it is not clear from our results whether earlier thymic engraftment by proT cells facilitated the subsequent wave of TSPs from the GFP^+^ TDBM donor graft, as we had postulated earlier (27). As such, for both the young and aged thymus, the initial delay in T cell cellularity seen at D14 when given only TDBM cells, is replaced by an equivalent or greater cellularity by D28 and beyond. This suggests that a delay in thymic crosstalk does not appear to significantly alter the recruitment capacity of the recovering thymus, despite the damage incurred by TECs due to the conditioning regimen.

Apart from lower cellularity, the thymuses of aged mice are quite distinct in their composition from their young counterparts, in that they showed a much larger contribution of host-derived cells. This may be due to the incomplete replacement of host-derived cells by GFP^+^ TDBM donor cells within the BM of aged mice, unlike their young counterparts. The ability of host cells to compete against the GFP^+^ graft and repopulate the BM conflicts with current literature showing many instances of aging cells being more susceptible to radiation exposure (38). One explanation could be that host cells in aged mice are being protected from the effects of radiation simply due to their higher body weight, as aged mice typically weighed twice as much as their young counterpart. While we set the CTX dose according to weight, a similar increase in radiation dose according to weight may have led to irreparable cellular damage. Nevertheless, mouse weight and size, as well as other metabolic differences, may add to the complexity in comparing the effects of conditioning in aged and young mice.

Future clinical applications of our findings will rely on the recent replacement of the xenogenic OP9-DL cells with a serum-free, cell-free system of plate-bound Dll4 or DL4-µbeads methods for the generation of human proT cells (39–41), which have increased the potential therapeutic use of proT cells. In addition, our findings strongly suggest that proTs could provide an immune boost to the elderly, the population that comprises the majority of patients undergoing HSCT. The next hurdles remaining before the therapeutic use of proT cells appear surmountable, though wide-ranging. Focusing on the preclinical side, it is paramount to demonstrate that proT-derived mature T cells can confer immunity against diseases in aged mice. Further, there is the need to address the standard practices of care in hospitals during HSCT and use them as a guide for our preclinical modelling. This includes details such as administration of both chemo- and radiation treatments, which we have done here, but also including anti-thymoglobulin (ATG) following HSCT (42) or the use of allogeneic grafts instead of congenic HSPCs. ATG treatment, for example, is given to curtail host vs graft rejection and other complications of HSCT, a practice that would likely counter proT cell therapy, as these cells would also be targeted by ATG. Thus, it requires further consideration before embarking on clinical trials.

In short, here we have improved upon the conditioning regimen and discovered that providing proT cells allows for the effective reconstitution of the aged mouse thymus with accelerated T cell regeneration. The favorable consequences afforded by rapid thymic reconstitution includes the appearance of mature T cells in secondary immune organs, providing a potential advantageous immune boost to aged recipients.

## Data Availability Statement

The raw data supporting the conclusions of this article will be made available by the authors, without undue reservation.

## Ethics Statement

The animal study was reviewed and approved by Sunnybrook Health Sciences Centre Animal Care Committee.

## Author Contributions

MM, YRL and CL performed all the experiments. MM and YRL designed and analyzed the data, and wrote the manuscript. JCZ-P. conceived the project, analyzed the data, and wrote and edited the manuscript. All authors contributed to the article and approved the submitted version.

## Funding

This work was supported by grants from the Canadian Institutes of Health Research (CHIR, FND-154332), Canadian Cancer Society Research Institute (No. 705960), Cancer Research Institute (CRI3872), and Stem Cell Network (SCN Ref: FY21/ACCT2-18), and National Institutes of Health (1R01HL147584-01A1). JCZ-P is supported by a Canada Research Chair in Developmental Immunology.

## Conflict of Interest

The authors declare that the research was conducted in the absence of any commercial or financial relationships that could be construed as a potential conflict of interest.

## Publisher’s Note

All claims expressed in this article are solely those of the authors and do not necessarily represent those of their affiliated organizations, or those of the publisher, the editors and the reviewers. Any product that may be evaluated in this article, or claim that may be made by its manufacturer, is not guaranteed or endorsed by the publisher.

## References

[1] Shortman K Wu L. Early T Lymphocyte Progenitors. Annu Rev Immunol (1996) 14:29–47. doi: 10.1146/annurev.immunol.14.1.29 8717506

[2] Goldschneider I. Cyclical Mobilization and Gated Importation of Thymocyte Progenitors in the Adult Mouse: Evidence for a Thymus-Bone Marrow Feedback Loop. Immunol Rev (2006) 209:58–75. doi: 10.1111/j.0105-2896.2006.00354.x 16448534

[3] Belteki G Haigh J Kabacs N Haigh K Sison K Costantini F. Conditional and Inducible Transgene Expression in Mice Through the Combinatorial Use of Cre-Mediated Recombination and Tetracycline Induction. Nucleic Acids Res (2005) 33:e51. doi: 10.1093/nar/gni051 15784609 PMC1069131

[4] Stadtfeld M Graf T. Assessing the Role of Hematopoietic Plasticity for Endothelial and Hepatocyte Development by Non-Invasive Lineage Tracing. Development (2005) 132:203–13. doi: 10.1242/dev.01558 15576407

[5] Petrie HT Zuniga-Pflucker JC. Zoned Out: Functional Mapping of Stromal Signaling Microenvironments in the Thymus. Annu Rev Immunol (2007) 25:649–79. doi: 10.1146/annurev.immunol.23.021704.115715 17291187

[6] Velardi E Tsai JJ van den Brink MRM. T Cell Regeneration After Immunological Injury. Nat Rev Immunol (2021) 21:277–91. doi: 10.1038/s41577-020-00457-z PMC7583557 33097917

[7] Verma R Foster RE Horgan K Mounsey K Nixon H Smalle N. Lymphocyte Depletion and Repopulation After Chemotherapy for Primary Breast Cancer. Breast Cancer Res (2016) 18:10. doi: 10.1186/s13058-015-0669-x 26810608 PMC4727393

[8] Marr KA. Delayed Opportunistic Infections in Hematopoietic Stem Cell Transplantation Patients: A Surmountable Challenge. Hematol Am Soc Hematol Educ Program (2012) 2012:265–70. doi: 10.1182/asheducation.V2012.1.265.3800160 PMC4696052 23233590

[9] Fletcher AL Lowen TE Sakkal S Reiseger JJ Hammett MV Seach N. Ablation and Regeneration of Tolerance-Inducing Medullary Thymic Epithelial Cells After Cyclosporine, Cyclophosphamide, and Dexamethasone Treatment. J Immunol (2009) 183:823–31. doi: 10.4049/jimmunol.0900225 19564346

[10] Williams KM Mella H Lucas PJ Williams JA Telford W Gress RE. Single Cell Analysis of Complex Thymus Stromal Cell Populations: Rapid Thymic Epithelia Preparation Characterizes Radiation Injury. Clin Transl Sci (2009) 2:279–85. doi: 10.1111/j.1752-8062.2009.00128.x PMC2741333 19750208

[11] Small TN Avigan D Dupont B Smith K Black P Heller G. Immune Reconstitution Following T-Cell Depleted Bone Marrow Transplantation: Effect of Age and Posttransplant Graft Rejection Prophylaxis. Biol Blood Marrow Transplant (1997) 3:65–75. Accession Number: 9267666 9267666

[12] Gui J Zhu X Dohkan J Cheng L Barnes PF Su DM. The Aged Thymus Shows Normal Recruitment of Lymphohematopoietic Progenitors But has Defects in Thymic Epithelial Cells. Int Immunol (2007) 19:1201–11. doi: 10.1093/intimm/dxm095 17804689

[13] Reis MD Csomos K Dias LP Prodan Z Szerafin T Savino W. Decline of FOXN1 Gene Expression in Human Thymus Correlates With Age: Possible Epigenetic Regulation. Immun Ageing (2015) 12:18. doi: 10.1186/s12979-015-0045-9 26516334 PMC4625732

[14] Zook EC Krishack PA Zhang S Zeleznik-Le NJ Firulli AB Witte PL. Overexpression of Foxn1 Attenuates Age-Associated Thymic Involution and Prevents the Expansion of Peripheral CD4 Memory T Cells. Blood (2011) 118:5723–31. doi: 10.1182/blood-2011-03-342097 PMC3228493 21908422

[15] Cepeda S Griffith AV. Thymic Stromal Cells: Roles in Atrophy and Age-Associated Dysfunction of the Thymus. Exp Gerontol (2018) 105:113–7. doi: 10.1016/j.exger.2017.12.022 PMC5869099 29278750

[16] Griffith AV Venables T Shi J Farr A van Remmen H Szweda L. Metabolic Damage and Premature Thymus Aging Caused by Stromal Catalase Deficiency. Cell Rep (2015) 12:1071–9. doi: 10.1016/j.celrep.2015.07.008 PMC4797338 26257169

[17] Hester AK Semwal MK Cepeda S Xiao Y Rueda M Wimberly K. Redox Regulation of Age-Associated Defects in Generation and Maintenance of T Cell Self-Tolerance and Immunity to Foreign Antigens. Cell Rep (2022) 38:110363. doi: 10.1016/j.celrep.2022.110363 35172147 PMC8898380

[18] Aw D Silva AB Maddick M von Zglinicki T Palmer DB. Architectural Changes in the Thymus of Aging Mice. Aging Cell (2008) 7:158–67. doi: 10.1111/j.1474-9726.2007.00365.x 18241323

[19] Dixit VD. Thymic Fatness and Approaches to Enhance Thymopoietic Fitness in Aging. Curr Opin Immunol (2010) 22:521–8. doi: 10.1016/j.coi.2010.06.010 PMC2993497 20650623

[20] Desanti GE Cowan JE Baik S Parnell SM White AJ Penninger JM. Developmentally Regulated Availability of RANKL and CD40 Ligand Reveals Distinct Mechanisms of Fetal and Adult Cross-Talk in the Thymus Medulla. J Immunol (2012) 189:5519–26. doi: 10.4049/jimmunol.1201815 PMC3605790 23152561

[21] Hikosaka Y Nitta T Ohigashi I Yano K Ishimaru N Hayashi Y. The Cytokine RANKL Produced by Positively Selected Thymocytes Fosters Medullary Thymic Epithelial Cells That Express Autoimmune Regulator. Immunity (2008) 29:438–50. doi: 10.1016/j.immuni.2008.06.018 18799150

[22] Rossi SW Kim MY Leibbrandt A Parnell SM Jenkinson WE Glanville SH. RANK Signals From CD4(+)3(-) Inducer Cells Regulate Development of Aire-Expressing Epithelial Cells in the Thymic Medulla. J Exp Med (2007) 204:1267–72. doi: 10.1084/jem.20062497 PMC2118623 17502664

[23] Singh J Mohtashami M Anderson G Zuniga-Pflucker JC. Thymic Engraftment by *In Vitro*-Derived Progenitor T Cells in Young and Aged Mice. Front Immunol (2020) 11:1850. doi: 10.3389/fimmu.2020.01850 32973763 PMC7462002

[24] Smith MJ Reichenbach DK Parker SL Riddle MJ Mitchell J Osum KC. T Cell Progenitor Therapy-Facilitated Thymopoiesis Depends Upon Thymic Input and Continued Thymic Microenvironment Interaction. JCI Insight (2017) 2:92056. doi: 10.1172/jci.insight.92056 28515359 PMC5436538

[25] Zakrzewski JL Kochman AA Lu SX Terwey TH Kim TD Hubbard VM. Adoptive Transfer of T-Cell Precursors Enhances T-Cell Reconstitution After Allogeneic Hematopoietic Stem Cell Transplantation. Nat Med (2006) 12:1039–47. doi: 10.1038/nm1463 16936725

[26] Zakrzewski JL Suh D Markley JC Smith OM King C Goldberg GL. Tumor Immunotherapy Across MHC Barriers Using Allogeneic T-Cell Precursors. Nat Biotechnol (2008) 26:453–61. doi: 10.1038/nbt1395 PMC2731996 18376399

[27] Awong G Singh J Mohtashami M Malm M La Motte-Mohs RN Benveniste PM. Human proT-Cells Generated *In Vitro* Facilitate Hematopoietic Stem Cell-Derived T-Lymphopoiesis *In Vivo* and Restore Thymic Architecture. Blood (2013) 122:4210–9. doi: 10.1182/blood-2012-12-472803 PMC5527400 24215033

[28] Mohtashami M Brauer PB Zúñiga-Pflücker JC. Induction of Human T Cell Development *In Vitro* With OP9-DL4-7fs Cells Expressing Human Cytokines. Methods Mol Biol (2022) in press. 10.1007/978-1-0716-2740-2_15 36374462

[29] Hensel JA Khattar V Ashton R Ponnazhagan S. Characterization of Immune Cell Subtypes in Three Commonly Used Mouse Strains Reveals Gender and Strain-Specific Variations. Lab Invest (2019) 99:93–106. doi: 10.1038/s41374-018-0137-1 30353130 PMC6524955

[30] Pinchuk LM Filipov NM. Differential Effects of Age on Circulating and Splenic Leukocyte Populations in C57BL/6 and BALB/c Male Mice. Immun Ageing (2008) 5:1. doi: 10.1186/1742-4933-5-1 18267021 PMC2268915

[31] Finn LE Foran JM. Are We Curing More Older Adults With Acute Myeloid Leukemia With Allogeneic Transplantation in CR1? Curr Opin Hematol (2016) 23:95–101. doi: 10.1097/MOH.0000000000000220 26825695

[32] Ogonek J Kralj Juric M Ghimire S Varanasi PR Holler E Greinix H. Immune Reconstitution After Allogeneic Hematopoietic Stem Cell Transplantation. Front Immunol (2016) 7:507. doi: 10.3389/fimmu.2016.00507 27909435 PMC5112259

[33] Gehre N Nusser A von Muenchow L Tussiwand R Engdahl C Capoferri G. A Stromal Cell Free Culture System Generates Mouse Pro-T Cells That can Reconstitute T-Cell Compartments In Vivo. Eur J Immunol (2015) 45:932–42. doi: 10.1002/eji.201444681 25408420

[34] Heng TS Goldberg GL Gray DH Sutherland JS Chidgey AP Boyd RL. Effects of Castration on Thymocyte Development in Two Different Models of Thymic Involution. J Immunol (2005) 175:2982–93. doi: 10.4049/jimmunol.175.5.2982 16116185

[35] Lepletier A Hun ML Hammett MV Wong K Naeem H Hedger M. Interplay Between Follistatin, Activin A, and BMP4 Signaling Regulates Postnatal Thymic Epithelial Progenitor Cell Differentiation During Aging. Cell Rep (2019) 27:3887–3901 e4. doi: 10.1016/j.celrep.2019.05.045 31242421

[36] Lynch HE Goldberg GL Chidgey A Van den Brink MR Boyd R Sempowski GD. Thymic Involution and Immune Reconstitution. Trends Immunol (2009) 30:366–73. doi: 10.1016/j.it.2009.04.003 PMC2750859 19540807

[37] Thoman ML. The Pattern of T Lymphocyte Differentiation is Altered During Thymic Involution. Mech Ageing Dev (1995) 82:155–70. doi: 10.1016/0047-6374(95)01597-S 8538244

[38] Hernandez L Terradas M Camps J Martin M Tusell L Genesca A. Aging and Radiation: Bad Companions. Aging Cell (2015) 14:153–61. doi: 10.1111/acel.12306 PMC4364827 25645467

[39] Reimann C Six E Dal-Cortivo L Schiavo A Appourchaux K Lagresle-Peyrou C. Human T-Lymphoid Progenitors Generated in a Feeder-Cell-Free Delta-Like-4 Culture System Promote T-Cell Reconstitution in NOD/SCID/gammac(-/-) Mice. Stem Cells (2012) 30:1771–80. doi: 10.1002/stem.1145 PMC3531890 22689616

[40] Shukla S Langley MA Singh J Edgar JM Mohtashami M Zuniga-Pflucker JC. Progenitor T-Cell Differentiation From Hematopoietic Stem Cells Using Delta-Like-4 and VCAM-1. Nat Methods (2017) 14:531–8. doi: 10.1038/nmeth.4258 28394335

[41] Trotman-Grant AC Mohtashami M De Sousa Casal J Martinez EC Lee D Brauer PM. DL4-μbeads Induce T Cell Differentiation From Stem Cells in a Stromal Cell-Free System. Nat Commun (2020) 12:5023. doi: 10.1038/s41467-021-25245-8 PMC8373879 34408144

[42] Novitzky-Basso I Remberger M Chen C Ellison C Pasic I Lam W. Anti-Thymocyte Globulin and Post-Transplant Cyclophosphamide do Not Abrogate the Inferior Outcome Risk Conferred by Human Leukocyte Antigen-A and -B Mismatched Donors. Eur J Haematol (2022) 108:288–97. doi: 10.1111/ejh.13735 34905239

