# Supplementary figures and images for: Thymus Reconstitution in Young and Aged Mice Is Facilitated by In Vitro-Generated Progenitor T Cells
Source: Front Immunol. 2022 Jul 8;13:926773. doi: 10.3389/fimmu.2022.926773 (PMC9304753; doi:10.3389/fimmu.2022.926773)

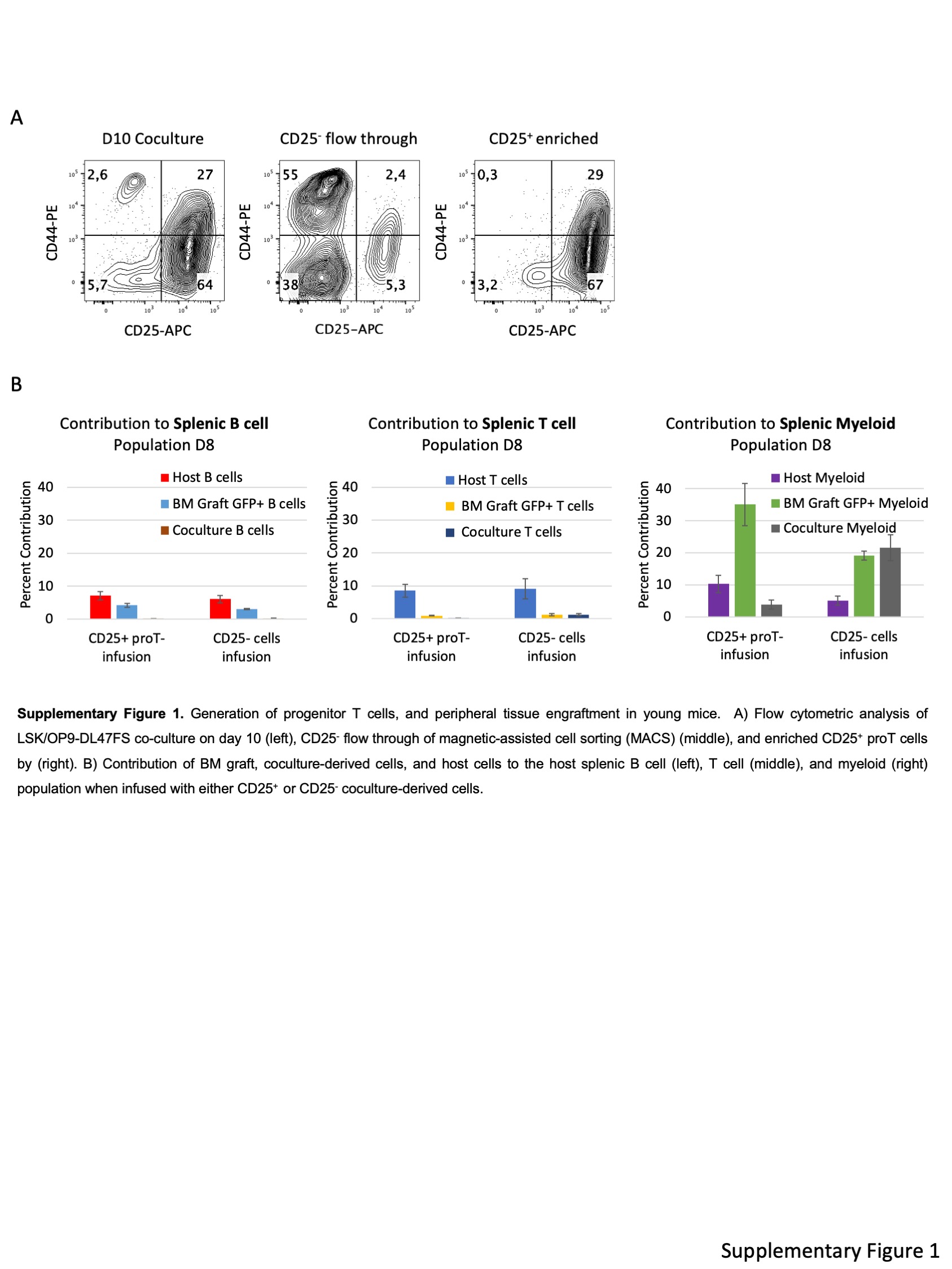

Supplement: Supplementary file 1 [file Image_1.jpeg]
